# Expression of concern: Quantum correlations are weaved by the spinors of the Euclidean primitives

**DOI:** 10.1098/rsos.190530

**Published:** 2019-04-10

**Authors:** The Royal Society Open Science Editorial Team

*R. Soc. open sci.* **5**, 180526 Published Online 30 May 2018 (doi:10.1098/rsos.180526)

Following the publication of ‘Quantum correlations are weaved by the spinors of the Euclidean primitives’ *R. Soc. open sci.* **5**, 180526 published 30 May 2018 (http://dx.doi.org/10.1098/rsos.180526), it has been brought to the attention of the Editorial team that there are concerns regarding this study.

A number of readers have written privately via email and also in extensive public correspondence, which is available via the ‘Comments’ section of the original article, raising a number of concerns regarding fundamental aspects of the work.

An investigation into these aspects is under way, and the journal is therefore issuing an expression of concern and will notify readers as to the results of our investigation as soon as possible.

