# Expression of Concern following post-publication assessment of Quantum correlations are weaved by the spinors of the Euclidean primitives

**DOI:** 10.1098/rsos.201777

**Published:** 2020-10-21

**Authors:** The Royal Society Open Science Editorial Team

*R. Soc. Open Sci.* **3**, 180526 (Published 30 May 2018) (doi:10.1098/rsos.180526)

Following peer review and publication of https://royalsocietypublishing.org/doi/10.1098/rsos.180526, *Royal Society Open Science* received correspondence expressing concern about the technical accuracy of this paper. Consequently, the journal conducted an independent *post hoc* assessment of the paper, resulting in this Expression of Concern.

A short explanation for the decision-making process to help readers understand why the editors opted to accept the paper is as follows:

1. As can be seen in the open review history of the paper, there was a divergence of opinion from the initial reviewers and the author mounted a response in a scholarly and comprehensive manner. This presents a challenge to editors: do the concerns outweigh potential positives? Does publishing a controversial approach add value to the literature through stimulating open debate? As for most academic journals, while reviewers make recommendations, editors make the final decision on what is acceptable for publication. Editors possess the ultimate veto. On balance, in this case, the decision was made to publish to encourage debate, which is central to scientific research.
2. While recognizing that a controversial paper may eventually be shown to contain flaws, it may be argued that all science is a work in progress. We move forward not in a linear process, but often in fractures of existing paradigms—the history of science is littered with examples of this. The editors felt that research presented in this paper may stimulate further debate and experimentation in this space. If a journal only ever published papers that reinforced existing mainstream knowledge, then science would be the poorer.
3. *Royal Society Open Science* has a remit to publish research that is appropriate for publication and which may be difficult to publish in more traditional journals. At the time of assessment and publication, the paper passed that test in the view of the editors. Furthermore, the journal's open peer review model allows readers to explore the review history for themselves.
4. Independent assessors that subsequently performed post-publication peer review on this manuscript have agreed to make their commentary available publicly and these are now online together with the author's responses. These comments can be read alongside this Expression of Concern. As the reader may note, there is no consensus on this work, which is why the journal has opted not to retract.
5. As with any human endeavour, the decision to publish a paper is not an exact science and editors balance a number of factors to decide what is appropriate. We remind readers that the journal permits and actively encourages replication studies. If readers with an interest in this field would like to submit either a replication or rebuttal of the author's work, we would welcome it.
6. It should be noted that correspondents critiquing the decision to publish this paper have been offered the opportunity to publish a rebuttal but, to date, have thus far declined to do so. We would once again encourage them and any others who may be interested to make open contributions in order to openly articulate and formalize the technical issues.

## Supplementary Material

Reviewer comments

