# Supplementary material for: Expression of Concern following post-publication assessment of Quantum correlations are weaved by the spinors of the Euclidean primitives
Source: R Soc Open Sci. 2020 Oct 21;7(10):201777. doi: 10.1098/rsos.201777 (PMC7657884; doi:10.1098/rsos.201777)
Supplement: Reviewer comments [file rsos201777_review_history.pdf]

THE ROYAL SOCIETY
PUBLISHING

ROYAL SOCIETY
OPEN SCIENCE

# Expression of Concern following post-publication assessment of Quantum correlations are weaved by the spinors of the Euclidean primitives

The Royal Society Open Science Editorial Team

**Article citation details**
*R. Soc. open sci.* **7**: 201777.
http://dx.doi.org/10.1098/rsos.201777

**Review timeline**
Original submission: 5 October 2020
Final acceptance: 5 October 2020

Note: Reports are unedited and appear as submitted by the referee. The review history appears in chronological order.

# Review History

## Decision letter (RSOS-201777.R0)

We hope you are keeping well at this difficult and unusual time. We continue to value your support of the journal in these challenging circumstances. If Royal Society Open Science can assist you at all, please don't hesitate to let us know at the email address below.

Dear Dr Open Science,

I am pleased to inform you that your manuscript entitled "Expression of Concern following post-publication assessment of Quantum correlations are weaved by the spinors of the Euclidean primitives" is now accepted for publication in Royal Society Open Science.

You can expect to receive a proof of your article in the near future. Please contact the editorial office (openscience_proofs@royalsociety.org) and the production office (openscience@royalsociety.org) to let us know if you are likely to be away from e-mail contact -- if you are going to be away, please nominate a co-author (if available) to manage the proofing process, and ensure they are copied into your email to the journal.

Due to rapid publication and an extremely tight schedule, if comments are not received, your paper may experience a delay in publication.

Royal Society Open Science operates under a continuous publication model. Your article will be published as soon as it is ready for publication, and this will be the final version of the paper. As

Reports © 2020 The Reviewers; Decision Letters © 2020 The Reviewers and Editors; Responses © 2020 The Reviewers, Editors and Authors. Published by the Royal Society under the terms of the Creative Commons Attribution License http://creativecommons.org/licenses/by/4.0/, which permits unrestricted use, provided the original author and source are credited

such, it can be cited immediately by other researchers. As the issue version of your paper will be the only version to be published I would advise you to check your proofs thoroughly as changes cannot be made once the paper is published.

Please see the Royal Society Publishing guidance on how you may share your accepted author manuscript at https://royalsociety.org/journals/ethics-policies/media-embargo/.

On behalf of the Editors of Royal Society Open Science, we look forward to your continued contributions to the Journal.

Kind regards,
Royal Society Open Science Editorial Office
Royal Society Open Science
openscience@royalsociety.org

Follow Royal Society Publishing on Twitter: @RSocPublishing
Follow Royal Society Publishing on Facebook:
https://www.facebook.com/RoyalSocietyPublishing.FanPage/
Read Royal Society Publishing's blog:
https://royalsociety.org/blog/blogsearchpage/?category=Publishing

## Author's Response to Decision Letter for (RSOS-201777.R0)

See Appendices A - E.

# Appendix A

## 1. Some problems with a recent Joy Christian paper

In 2018, a paper was published in the Royal Society *Open Science* journal by Joy Christian ([1]), called 'Quantum correlations are weaved by the spinors of the Euclidean primitives'. This makes some strong claims about Bell's Theorem in quantum mechanics, and its relation to the sphere $S^7$ and the exceptional group $E_8$. Perhaps most startling mathematically, as against physically, however, is the author's claim to have discovered a new *associative* version of the normed division algebra hitherto represented by the octonians. Joy Christian uses Geometric Algebra in his work, and claims that his new physical results in Quantum Mechanics stem from the employment of GA, and the additional geometrical elements of reality which it can introduce in addition to the usual complex numbers used in Quantum Mechanics. In previous papers he has mainly considered the ordinary GA, but in the 2018 Royal Society paper he says he is considering the 'Conformal Geometric Algebra' and explicitly links the 'Euclidean primitives' of CGA with his statements about Bell's Theorem.

Christian's work has repeatedly been criticised mathematically, but he has several times stated that no one well-versed in Geometric Algebra has explicitly criticsed his mathematics in print, and that this suggests his critics simply do not understand the GA in his work, not that his mathematics is wrong. I have looked at the first sections of the Royal Society paper, and found that the CGA he is using is a version of what is known as 'the 1d-up approach', which adds just one additional vector, with non-zero square to the basis vectors of $\mathbb{E}^3$, rather than the two additional vectors with zero square used in the usual CGA (e.g. of the type introduced by Hestenes [2]). This is fine, however, there are many demonstrable mathematical errors in the work which renders most of what is said about the properties of this '1d up' CGA invalid. These will be summarised shortly. However, it is very necessary to stress that this in no way is meant to transfer through to being a comment about what Christian is saying as regards Bell's Theorem. This may or may not have any substance to it, and at a certain level there is certainly an interest in taking a 1d up CGA approach to quantum mechanics and electromagnetism, as e.g. discussed briefly in a relativistic context in [3]. In particular the comments made here only relate to Sections 1 and 2 of the Christian paper, before the main work on Bell's Theorem begins. However the mathematical errors and misstatements in this first part of the paper would certainly need to be remedied before being able to approach the second part properly, hence it seems of value to record the objections here. Note several of the points made here have been made independently by Richard D. Gill and others in the discussion thread attached to the Royal Society paper: `https://royalsocietypublishing.org/doi/full/10.1098/rsos.180526#disqus_thread`, but what may be useful here is decoding what Christian is claiming in terms of the unexpected link with the 1d up approach, and also making the first statements on these issues from a practicing GA person (which may or may not be helpful!).

### 1.1. Initial problems

(Note most equation numbers from now on relate to those in the paper ([1]), and we will say explicitly if we mean an equation in the current contribution.)

The first maths problem comes in Christian's equation (2.20). which says

$$e_\infty^2 = 0 \tag{1.1}$$

and then that

> 'Such a vector that is orthogonal to itself is called a null vector in Conformal Geometric Algebra [18]. It is introduced to represent both finite points in space as well as points at infinity [19]. As points thus defined are null-dimensional or dimensionless, addition of $e_\infty$ into the algebraic structure of $\mathbb{E}^3$ does not alter the latter's dimensions but only its point-set topology, rendering it diffeomorphic to a closed, compact, simply connected 3-sphere ... '

I do not see how it can be thought introducing $e_\infty$ into the algebraic structure of $\mathbb{E}^3$ does not alter its dimensions. Also, since null vectors are described as representing both finite points as well as points at infinity, which is true in the CGA, the logic of this paragraph seems to be that since these points are null-dimensional or dimensionless then addition of any of them into $\mathbb{E}^3$ could go ahead and $\mathbb{E}^3$ would only be changed in topology.

The main problem with (2.20), however, is that it is shortly contradicted by (2.32), which says that $e_\infty^2 = 1$. To give the full context at this point, it is said:

> 'The three-dimensional physical space — i.e. the compact 3-sphere we discussed above — can now be viewed as embedded in the four-dimensional ambient space, $\mathbb{R}^4$, as depicted in figure 2. In this higher dimensional space, $e_\infty$ is then a unit vector,
>
> $$||e_\infty^2|| = e_\infty \cdot e_\infty = 1 \iff e_\infty^2 = 1$$
>
> and the corresponding algebraic representation space (2.31) is nothing but the eight-dimensional even sub-algebra of the 24 =16-dimensional Clifford algebra $Cl_{4,0}$. Thus, a one-dimensional subspace — represented by the unit vector $e_\infty$ in the ambient space $\mathbb{R}^4$ — represents a null-dimensional space — i.e. the infinite point of $\mathbb{E}^3$ — in the physical space $S^3$.'

Again this is not comprehensible mathematically, and indeed anything which tries to reconcile $e_\infty^2 = 0$ in 3d, with the same object squaring to +1 when interpreted as living in 4d, is going to be problematical. I think what is going on is that Christian is trying to use not the usual Conformal Geometric Algebra, in which the additional basis vectors square to zero, but the 1d up CGA, as discussed e.g. in [4] or [3]. Here, the base Euclidean space is extended by 1 additional vector, squaring to plus or minus 1, and thus

becomes either a spherical or hyperbolic space. This shares the key property of the usual CGA that translations can be carried out by rotors, but with the key difference that we are now working in a *constant curvature space*, so that in an application to ordinary 3d Euclidean space we would need to make the radius of curvature of the space large enough for it not to matter to the results, if we wanted to preserve all the standard Euclidean relations under translation. The paper [4] gives a detailed description of the application of these methods to rigid body mechanics in 3d, and the key point here is that the space described there by augmenting the three Euclidean basis vectors $e_i$ all squaring to $+1$, by an additional vector $e$, also squaring to $+1$, is *identical* to the space being described in the Christian paper in which (eventually), the added vector, here called $e_\infty$, squares to $+1$. Note this combination of signs means we are dealing with a spherical rather than hyperbolic space.

So we can understand what Christian is trying to say in the two paragraphs we have picked out so far, as that he is going to use a '1d up' version of CGA, in which the base space points are going to be represented by unit vectors in the higher space, lying on the surface of a sphere, and which can therefore be moved around by the rotors of $Cl_{4,0}$. The extra dimension we need to introduce for this is defined by a basis vector $e_\infty$ squaring to $+1$, and there is never any question of its squaring to 0.

The next problem is equation (2.25), which concerns the reversion properties of the pseudoscalar $I_c$, which is introduced in equation (2.24) as

$$I_c = e_x e_y e_z e_\infty \tag{1.2}$$

The multiplication properties given for the 8 quantities $\{1, e_x e_y, e_z e_x, e_y e_z, e_x e_\infty, e_y e_\infty, e_z e_\infty, I_c\}$ in Table 1, tells us unambiguously that these quantities correspond to the 8 elements of the even subalgebra of $Cl_{4,0}$, with $I_c$ being the pseudoscalar for this space. This is not different from what Christian says, but he says in equation (2.25) that $I_c$ reverses to minus itself, i.e. (to quote)

$$I_c^\dagger = I_3^\dagger e_\infty = -I_3 e_\infty = -I_c \tag{1.3}$$

(Note there appears to be no dispute over the dagger operation being 'reversion', and we will denote it with the usual tilde from now on.)

But this equation is *wrong*. We have

$$\tilde{I}_c = e_\infty e_z e_y e_x = -e_z e_y e_x e_\infty = -e_y e_x e_z e_\infty = e_x e_y e_z e_\infty = I_c \tag{1.4}$$

i.e. it reverses to *plus* itself. Thus if Christian's equation (2.25) is used anywhere, it will lead to error.

The next problem is with equations (2.33) and (2.34), which read

$$\begin{aligned} \mathcal{K}^+ &= \text{span}\{1, e_x e_y, e_z e_x, e_y e_z, e_x e_\infty, e_y e_\infty, e_z e_\infty, I_c\} \\ \mathcal{K}^- &= \text{span}\{1, -e_x e_y, -e_z e_x, -e_y e_z, -e_x e_\infty, -e_y e_\infty, -e_z e_\infty, -I_c\} \end{aligned} \tag{1.5}$$

It seems to be important to Christian's later purposes that $\mathcal{K}^+$ and $\mathcal{K}^-$ are different, but as spans of objects which differ just by scalar factors from the same objects in the other set, they are mathematically identical. Presumably Christian means something different from what he has written,

but this would have to be explained, using actual mathematics, before one could continue with the paper.

(Continuing anyway!) the next problem is with the title and initial remarks of Section 2.4 in the Christian paper. The title is 'Representation space $\mathcal{K}^\lambda$ remains closed under multiplication' and the initial remarks are 'As an eight-dimensional linear vector space, $K^\lambda$ has some remarkable properties. To begin with, $K^\lambda$ is closed under multiplication.' The title and remarks seem naive — we are dealing with the even subset of the Clifford algebra $Cl_{4,0}$ so what is said here follows immediately from this fact. The properties are hardly remarkable *per se.*

More serious is what happens next. It is clear from Christian's equation (2.8) that by 'norm' of a general multivector $M$ he means

$$||M||= \sqrt{\langle M\tilde{M}\rangle} \tag{1.6}$$

We can see that this square root is valid, and won't lead to imaginaries, as follows. Let us set up a general $M$ via defining two 2-spinors $\phi$ and $\chi$ as

$$\begin{aligned} \phi &= a_0 + a_1 e_y e_z + a_2 e_z e_x + a_3 e_x e_y \\ \chi &= b_0 + b_1 e_y e_z + b_2 e_z e_x + b_3 e_x e_y \end{aligned} \tag{1.7}$$

and write

$$M = \phi + I\chi \tag{1.8}$$

(Note we are going to write $I_c$ as $I$ from now on). Since $I^2 = 1$ we have

$$M\tilde{M} = \phi\tilde{\phi} + \chi\tilde{\chi} + I\left(\phi\tilde{\chi} + \chi\tilde{\phi}\right) \tag{1.9}$$

Now, let us define two 4-vectors using the components of $\phi$ and $\chi$

$$\begin{aligned} a &= a_0 e_\infty + a_1 e_1 + a_2 e_2 + a_3 e_3 \\ b &= b_0 e_\infty + b_1 e_1 + b_2 e_2 + b_3 e_3 \end{aligned} \tag{1.10}$$

(Note we are not saying that $\phi$ or $\chi$ are 4-vectors. We are just defining objects that make it easy to display the components of $M\tilde{M}$.) Then we find $\phi\tilde{\phi} + \chi\tilde{\chi} = a^2 + b^2$ and $\phi\tilde{\chi} + \chi\tilde{\phi}$ is the scalar $2a{\cdot}b$, meaning

$$M\tilde{M} = a^2 + b^2 + 2a{\cdot}bI \tag{1.11}$$

This shows us that $\langle M\tilde{M}\rangle = a^2 + b^2$ is indeed positive if $M$ is non-zero, hence the norm is well-defined.

Given any two general elements $X$ and $Y$, Christian then decides to normalise them, setting

$$||X||^2 = 1, \quad ||Y||^2 = 1 \tag{1.12}$$

Christian at this point does not motivate why we would wish to do this, but as just established, it is something we can indeed carry out for any non-zero elements.

So far, so good. However, things go very wrong with equation (2.40). Christian states:

> We shall soon see that for vectors $X$ and $Y$ in $K^\lambda$ (not necessarily unit) the following relation holds:
>
> $$||XY|| = ||X||\,||Y|| \qquad (2.40)$$

(By 'vector' Christian means what we would call 'multivector' here, as is clear from the context.) However, this is false. Consider the quantities

$$I_+ = \frac{1}{2}(1+I), \quad I_- = \frac{1}{2}(1-I) \tag{1.13}$$

Since $I$ squares to 1 and is its own reverse, then these satisfy the relations

$$I_+^2 = I_+\tilde{I}_+ = I_+, \quad I_-^2 = I_-\tilde{I}_- = I_-, \quad I_+I_- = I_-I_+ = 0 \tag{1.14}$$

We call such quantities 'idempotents' (since they square to themselves) and this particular pair are 'orthogonal' (since their product is zero). Now let

$$X = \sqrt{2}I_+, \quad Y = \sqrt{2}I_- \tag{1.15}$$

These satisfy

$$||X|| = 1, \quad ||Y|| = 1, \quad \text{but} \quad ||XY|| = 0 \tag{1.16}$$

This disproves the assertion in Christian's (2.40). It also means that the assertion which follows it:

> One of the important observations here is that, without loss of generality, we can restrict our representation space to a set of *unit* vectors in $K^\lambda$

is false, since if $||X||$ and $||Y||$ are unit vectors, it does *not* follow that $Z = XY$ is also a unit vector, despite what Christian says in his equation (2.41).

In Section 2.5 there is further confusion about a quantity which when first introduced squares to 0, but then later squares to 1. In (2.47) and (2.48) the quantity $\epsilon$, which satisfies $\epsilon^2 = 0$ is brought in to allow the definition of *biquaternions*, via

$$\mathbb{Q}_z = \boldsymbol{q}_r + \boldsymbol{q}_d\epsilon \tag{1.17}$$

where $\boldsymbol{q}_r$ and $\boldsymbol{q}_r$ are quaternions. In equation (2.51), however, $\epsilon$ is identified with $-I$, and it is stated that $\epsilon^2 = +1$. Thus the previous reference to biquaternions was a red herring. What is actually being introduced is the construction we have used above, where one can write a general element of the even subalgebra of $Cl_{(4,0)}$ as

$$M = \phi + I\chi \tag{1.18}$$

with $\phi$ and $\chi$ as given in equations (1.7). We called these 2-spinors above, but it is fine to identify them as quaternions as well. So this shows that translating the quantities introduced by Christian in this section into our notation, we have

$$\boldsymbol{q}_r = \phi, \quad \boldsymbol{q}_d = \chi, \quad \mathbb{Q}_z = \boldsymbol{q}_r + \boldsymbol{q}_d\epsilon = M = \phi + I\chi \tag{1.19}$$

(A slight problem is that since Christian says that $\epsilon$ is equal to the *reverse* of $I$ and he believes (wrongly) that this is $-I$, some signs will start to get out of drift as regards components of his $\boldsymbol{q}_d$ quaternion versus our $\chi$, but I don't think this is crucial.)

Now we have so far skipped over one feature of the construction of $\mathbb{Q}_z$, which is that Christian wants each of $\boldsymbol{q}_r$ and $\boldsymbol{q}_d$ to be *normalised*, with

$$||\boldsymbol{q}_r||=||\boldsymbol{q}_d||=\varrho \tag{1.20}$$

where $\varrho$ is some fixed scalar. He then correctly says in equation (2.53) that this means

$$||\mathbb{Q}_z||=\sqrt{2}\varrho \tag{1.21}$$

However, things go very wrong in the next equation. Christian says

> Now the normalization of $\mathbb{Q}_z$ in fact necessitates that every $\boldsymbol{q}_r$ be orthogonal to its dual $\boldsymbol{q}_d$
>
> $$||\mathbb{Q}_z||=\sqrt{2}\varrho \implies \boldsymbol{q}_r\tilde{\boldsymbol{q}}_d+\boldsymbol{q}_d\tilde{\boldsymbol{q}}_r=0 \tag{1.22}$$

This is false. The same result in the above notation (as used in our equation (1.11)) would be that

$$a=\varrho \quad \text{and} \quad b=\varrho \implies a{\cdot}b=0 \tag{1.23}$$

which is patently wrong. So can we understand why Christian believes this? Tracing through what happens in the following two equations, it is clear that the mistake is made at the point where he says that it is needed for $\mathbb{Q}_z\tilde{\mathbb{Q}}_z$ to be a scalar. If it *were* needed then indeed it follows that $2a{\cdot}b=\boldsymbol{q}_r\tilde{\boldsymbol{q}}_d+\boldsymbol{q}_d\tilde{\boldsymbol{q}}_r$ would have to vanish, but from what has been said about $\mathbb{Q}_z$ so far there is no such requirement — it has just been required that the norm (as defined by Christian and which we examined above) has to have value $2\varrho$. It looks as though what's happening is that Christian has temporarily forgotten that the norm is just the scalar part of the product $M\tilde{M}$, not both the scalar and grade-4 parts. This is a very important mistake.

Of course there had to be a significant mistake, since it is needed to be able to prove the (false) assertion about the product of the norms being the norm of the product. This is repeated in terms of $\mathbb{Q}$s in equation (2.59). The 'proof' of this amounts to the fact that if two $\mathbb{Q}$s each individually have vanishing grade-4 part when forming $\mathbb{Q}_z\tilde{\mathbb{Q}}_z$, then the product of their norms is equal to the norm of their product. This is fine, but only applies to this special class of $\mathbb{Q}$s, not the whole even subalgebra of $Cl_{(4,0)}$, as Christian claims.

There is a lot of discussion around this part of Section 2.5, attempting to say that due to the relations proved for the norms, therefore he has discovered a new *associative* version of the normed division algebra hitherto represented by the octonians, but of course this is absolutely false, as it had to be, since the relations he is talking about only apply to a limited subset of the space, not the whole space.

### 1.2. What has been achieved by the end of Section 2 of the Christian paper

This completes a quick survey of the initial problems in the Christian paper, taking us through to the start of the discussion concerning quantum

state. Hence this is a good time to set down what the mathematical apparatus Christian has assembled to this point actually amounts to. This can be summarised as follows.

Let us consider the even subalgebra of $Cl_{(4,0)}$ and pick out the elements $R$ from this which satisfy

$$R\tilde{R} = 1$$

i.e. we pick out the set of what are usually called *rotors* in this space. Then all of Christian's working to this point boils down to the result that if $S$ is another rotor, then the combination $SR$ is a further rotor, since it satisfies

$$SR\widetilde{(SR)} = SR\tilde{R}\tilde{S} = 1 \tag{1.24}$$

This is true but hardly very exciting!

## References


[1] Christian, J.: Quantum correlations are weaved by the spinors of the euclidean primitives. Royal Society Open Science **5**, 180526 (2018).

[2] Hestenes, D.: Old wine in new bottles: A new algebraic framework for computational geometry. In E Bayro-Corrochano, G. S. (Ed.), *Geometric Algebra with Applications in Science and Engineering*, 3–17 (Springer, 2001).

[3] Lasenby, A.: Recent applications of conformal geometric algebra. In *Computer Algebra and Geometric Algebra with Applications*, 298–328 (Springer, 2004).

[4] Lasenby, A.: Rigid body dynamics in a constant curvature space and the '1D-up' approach to conformal geometric algebra. In *Guide to Geometric Algebra in Practice*, 371–389 (Springer, 2011).

# Appendix B

**Published Article DOI**: https://doi.org/10.1098/rsos.180526

**Article Title**: Quantum Correlations are Weaved by the Spinors of the Euclidean Primitives

**Author**: Joy Christian

**To**: Prof. Andrew Dunn, Senior Publishing Editor, Royal Society Open Science

---

Dear Prof. Dunn,

Thank you for sending me additional reviewer comments on my published paper. Thank you also for giving me the opportunity to respond to these comments. Below please find my full, point-by-point response to the comments.

Kind regards,

Joy Christian

---

**Author's Response to the Additional Reviewer Comments**

**Reviewer: 1. Some problems with a recent Joy Christian paper**

In 2018, a paper was published in the Royal Society *Open Science* journal by Joy Christian ([1]), called 'Quantum correlations are weaved by the spinors of the Euclidean primitives'. This makes some strong claims about Bell's Theorem in quantum mechanics, and its relation to the sphere $S^7$ and the exceptional group $E_8$.

**Author response**: The primary goal of my paper is not Bell's theorem *per se* or the exceptional group $E_8$, but a geometrical explanation of the origins of quantum correlations, as correlations among the points of the 7-sphere constructed in the manuscript (cf. Theorem 3.1 on page 13). Moreover, Bell's theorem is not a theorem "in quantum mechanics", nor does it have any relation to either $S^7$ or $E_8$. Indeed, not a single concept of quantum theory is used in its proof. In fact, despite its name, it is not even a theorem in the mathematical sense but a physical argument based on a number of questionable assumptions. In my paper I have indeed questioned some of these assumptions [1].

But I am not the first or only physicist to have criticised Bell's theorem. Criticisms of Bell's theorem began soon after its publication, which included high-profile critics such as Louis de Broglie. They have never ceased ever since. During the intermediate years of its acceptance by the physics community, one of the ardent critics of Bell's theorem has been Arthur Fine, who pointed out as early as in 1980's that Bell's argument depends on considering joint probability distribution of three or four mutually incompatible experiments, which is not a justifiable assumption on physical grounds. By now there exists a vast literature on various criticisms of Bell's theorem. While not all such criticisms

are of high quality, there do exist a number of high quality criticisms, published in respected peer-review journals, by established scientists like Karl Hess, Itamar Pitowsky, Hans de Raedt and Andrei Khrennikov, to mention only a few.

**Reviewer:** Perhaps most startling mathematically, as against physically, however, is the author's claim to have discovered a new associative version of the normed division algebra hitherto represented by the octonians.

**Author response**: This is correct. For convenience, I have presented the mathematical contents of the said associtive normed division algebra in eight dimensions in the following preprint, entitled "Eight-dimensional Octonion-like but Associative Normed Division Algebra": `https://hal.archives-ouvertes.fr/hal-01933757/document`.

**Reviewer:** Joy Christian uses Geometric Algebra in his work, and claims that his new physical results in Quantum Mechanics stem from the employment of GA, and the additional geometrical elements of reality which it can introduce in addition to the usual complex numbers used in Quantum Mechanics.

**Author response**: This description of my work by the reviewer is not correct. The results found in my work using Geometric Algebra are not "in" quantum mechanics. That is to say, they are not results within the usual formalism of quantum mechanics. In fact, *no* quantum mechanical concept is used in my derivation of the strong (or "quantum") correlations using Geometric Algebra. It is therefore not correct to state as done by the reviewer that I claim that my "new physical results in Quantum Mechanics stem from the employment of GA, and the additional geometrical elements of reality which it can introduce in addition to the usual complex numbers used in Quantum Mechanics."

**Reviewer:** In previous papers he has mainly considered the ordinary GA, but in the 2018 Royal Society paper he says he is considering the 'Conformal Geometric Algebra' and explicitly links the 'Euclidean primitives' of CGA with his statements about Bell's Theorem.

**Author response**: This is correct. I have used a version of 'Conformal Geometric Algebra' in the 2018 RSOS paper.

**Reviewer:** Christian's work has repeatedly been criticised mathematically, but he has several times stated that no one well-versed in Geometric Algebra has explicitly criticsed his mathematics in print, and that this suggests his critics simply do not understand the GA in his work, not that his mathematics is wrong.

**Author response**: This is correct. No one with expertise or peer-reviewed publication record in geometric algebra has ever published a criticism of my work. In fact, some expertise or peer-reviewed publication record, not only in geometric algebra, but also in division algebras, differential geometry, topology, fiber bundles, and general relativity is essential for understanding my work on quantum correlations, as it relates to Bell's theorem. In addition to the Review History of my RSOS paper [1] and in online discussions on the Disqus, I have addressed all criticisms in the following five preprints: arXiv:quant-ph/0703244, arXiv:1110.5876, arXiv:1203.2529, arXiv:1301.1653 and arXiv:1501.03393. For a recent summary, see Ref. [3].

**Reviewer:** I have looked at the first sections of the Royal Society paper, and found that the CGA he is using is a version of what is known as 'the 1d up approach', which adds just one additional vector, with non-zero square to the

basis vectors of $\mathbb{E}^3$, rather than the two additional vectors with zero square used in the usual CGA (e.g. of the type introduced by Hestenes [2]).

**Author response**: This is essentially correct. However, there is more to what I have constructed in the RSOS paper. What is stated by the reviewer is the bare essential and it is an oversimplified view of my approach to quantum correlations.

**Reviewer:** This is fine, however, there are many demonstrable mathematical errors in the work which renders most of what is said about the properties of this '1d up' CGA invalid. These will be summarised shortly.

**Author response**: I respectfully disagree. My point-by-point response to the reviewer's objections follows below.

**Reviewer:** However, it is very necessary to stress that this in no way is meant to transfer through to being a comment about what Christian is saying as regards [to] Bell's Theorem. This may or may not have any substance to it, and at a certain level there is certainly an interest in taking a 1d up CGA approach to quantum mechanics and electromagnetism, as e.g. discussed briefly in a relativistic context in [3].

**Author response**: I would like to stress that what I have presented is not "a 1d up CGA approach to quantum mechanics" *per se*. What I have presented has an entirely different ontological status concerning quantum correlations.

**Reviewer:** In particular the comments made here only relate to Sections 1 and 2 of the Christian paper, before the main work on Bell's Theorem begins. However the mathematical errors and misstatements in this first part of the paper would certainly need to be remedied before being able to approach the second part properly, hence it seems of value to record the objections here.

**Author response**: I respectfully disagree. In what follows all of the objections raised by the reviewer are addressed in detail.

**Reviewer:** Note several of the points made here have been made independently by Richard D. Gill and others in the discussion thread attached to the Royal Society paper: `https://royalsocietypublishing.org/doi/full/10.1098/rsos.180526#disqus_thread`, ...

**Author response**: The same discussion thread also contains my detailed replies to each of the issues raised. What is more, on careful reading one will find that Richard D. Gill has in fact conceded to a number of key issues in that discussion thread.

**Reviewer:** ... but what may be useful here is decoding what Christian is claiming in terms of the unexpected link with the 1d up approach, and also making the first statements on these issues from a practicing GA person (which may or may not be helpful!).

**Author response**: It is not helpful to focus only on the "1d up approach" to Conformal Geometric Algebra. That is

not a helpful view of what I have presented in my paper. In fact, it is quite a narrow view. What I have identified in the paper is an algebraic representation space $S^7$ of the compactified physical space $S^3$. Now this algebraic representation space happens to be related to the "1d up approach" to CGA, but that is not the main message conveyed in my paper. It is a physics paper, not a mathematics paper. The physical concepts presented therein are vitally important.

**Reviewer:** 1.1. Initial problems

(Note most equation numbers from now on relate to those in the paper ([1]), and we will say explicitly if we mean an equation in the current contribution.)

**Reviewer:** The first maths problem comes in Christian's equation (2.20), which says

$$\mathbf{e}_\infty^2 = 0 \tag{1.1}$$

and then that

> 'Such a vector that is orthogonal to itself is called a null vector in Conformal Geometric Algebra [18]. It is introduced to represent both finite points in space as well as points at infinity [19]. As points thus defined are null-dimensional or dimensionless, addition of $\mathbf{e}_\infty$ into the algebraic structure of $\mathbb{E}^3$ does not alter the latter's dimensions but only its pointset topology, rendering it diffeomorphic to a closed, compact, simply connected 3-sphere . . . '

I do not see how it can be thought introducing $\mathbf{e}_\infty$ into the algebraic structure of $\mathbb{E}^3$ does not alter its dimensions. Also, since null vectors are described as representing both finite points as well as points at infinity, which is true in the CGA, the logic of this paragraph seems to be that since these points are null-dimensional or dimensionless then addition of any of them into $\mathbb{E}^3$ could go ahead and $\mathbb{E}^3$ would only be changed in topology.

**Author response**: There is no "maths problem" or even a conceptual problem here. What is stated in my paragraph quoted by the reviewer is quite easy to understand, unless one has missed what has been presented in the Subsection 2.1 entitled "One-point compactification of the three-dimensional Euclidean Space." In equation (2.15) of that subsection the null vector $\mathbf{e}_\infty$ is introduced, which represents a single dimensionless point at infinity in the physical space $\mathbb{E}^3$:

$$S^3 = \mathbb{E}^3 \cup \{\mathbf{e}_\infty\}\,. \tag{2.15}$$

This is an example of the well known one-point or Alexandroff compactification. Here $\mathbf{e}_\infty$ is introduced in Subsection 2.1 after considerable discussion, providing explicit construction in equations (2.12) to (2.14), and with the help of figures 2 and 3 (I have reproduced figure 2 on the next page for convenience). As we can see from figure 2, $\mathbf{e}_\infty$ is clearly a null vector [2] within the compactified physical space $S^3$, but a unit vector within the space $\mathbb{R}^4$ that embeds $S^3$. Thus the logic of my paragraph quoted by the reviewer is that the addition of the null vector $\mathbf{e}_\infty$ to $\mathbb{E}^3$ in the manner shown in figure 2 transforms $\mathbb{E}^3$ into $S^3$, as stated in equation (2.15). Moreover, it is clear that the spaces $\mathbb{E}^3$ and $S^3$ are both *three*-dimensional and differ only by a single dimensionless point, whereas the space $\mathbb{R}^4$ that embeds the

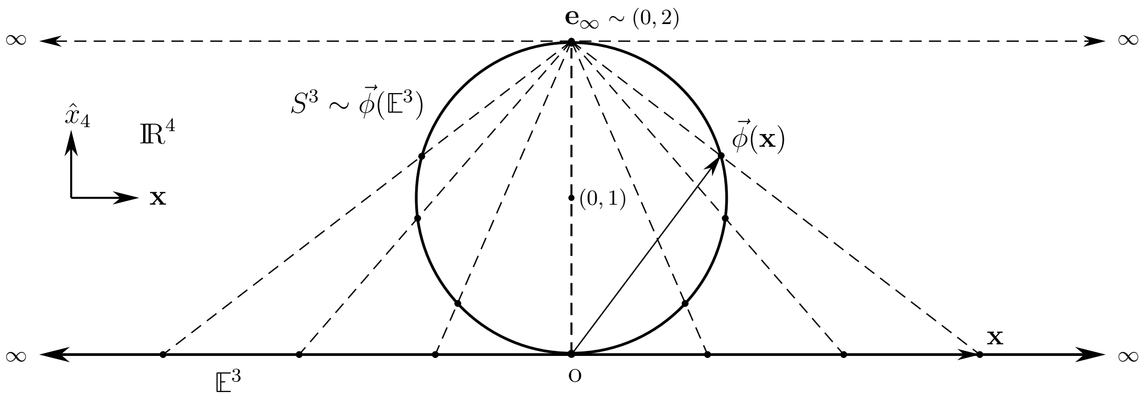


FIG. 1: One-point compactification of the Euclidean space $\mathbb{E}^3$ by means of a stereographic projection onto $S^3 \in \mathrm{I\!R}^4$.

space $S^3$ is four-dimensional. Thus the addition of the null vector $\mathbf{e}_\infty$ to $\mathbb{E}^3$ compactifies it into $S^3$, thereby changing its topology from $\mathrm{I\!R}^3$ to $S^3$, but retaining the algebraic structure of the space $\mathbb{E}^3$. Unfortunately, the reviewer seems to have overlooked Subsection 2.1 and jumped straight to the Subsection 2.2 containing equation (2.20).

**Reviewer:** The main problem with (2.20), however, is that it is shortly contradicted by (2.32), which says that $\mathbf{e}_\infty^2 = 1$. To give the full context at this point, it is said:

> 'The three-dimensional physical space — *i.e.*, the compact 3-sphere we discussed above — can now be viewed as embedded in the four-dimensional ambient space, $\mathrm{I\!R}^4$, as depicted in figure 2. In this higher dimensional space $\mathbf{e}_\infty$ is then a *unit* vector,
>
> $$||\mathbf{e}_\infty||^2 = \mathbf{e}_\infty \cdot \mathbf{e}_\infty = 1 \iff \mathbf{e}_\infty^2 = 1\,,$$
>
> and the corresponding algebraic representation space (2.31) is nothing but the eight-dimensional *even* sub-algebra of the $2^4 = 16$-dimensional Clifford algebra $Cl_{4,0}$. Thus a one-dimensional subspace — represented by the unit vector $\mathbf{e}_\infty$ in the ambient space $\mathrm{I\!R}^4$ — represents a *null*-dimensional space — *i.e.*, the infinite point of $\mathbb{E}^3$ — in the physical space $S^3$.'

Again this is not comprehensible mathematically, and indeed anything which tries to reconcile $e_\infty^2 = 0$ in 3d, with the same object squaring to +1 when interpreted as living in 4d, is going to be problematical.

**Author response**: There is nothing incomprehensible or problematical or unorthodox here. As noted in my previous response, there is no contradiction between equations (2.20) and (2.32). To be sure, these equations would have been contradictory if they had been claimed to be meaningful within same mathematical space. But that is not the case. It is evident from the one-point compactification illustrated in figure 2 that $\mathbf{e}_\infty$ is a null vector within $S^3$ but a unit vector within its embedding space $\mathrm{I\!R}^4$. Now the reviewer claims — *without demonstration* — that this "is going to be problematical." Where in the paper has it become problematical? It hasn't. The purpose of the one-point

compatification of the space $\mathbb{E}^3$ is physical, as discussed in the Subsection 2.1. The actual calculations of the strong correlations are carried out entirely using the algebra given in the multiplication table 1 on page 8 of the paper, which involves only $e_\infty^2 = 1$. Thus the difficulty suggested by the reviewer does not arise.

**Reviewer:** I think what is going on is that Christian is trying to use not the usual Conformal Geometric Algebra, in which the additional basis vectors square to zero, but the 1d up CGA, as discussed e.g. in [4] or [3]. Here, the base Euclidean space is extended by 1 additional vector, squaring to plus or minus 1, and thus becomes either a spherical or hyperbolic space. This shares the key property of the usual CGA that translations can be carried out by rotors, but with the key difference that we are now working in a *constant curvature space*, so that in an application to ordinary 3d Euclidean space we would need to make the radius of curvature of the space large enough for it not to matter to the results, if we wanted to preserve all the standard Euclidean relations under translation. The paper [4] gives a detailed description of the application of these methods to rigid body mechanics in 3d, and the key point here is that the space described there by augmenting the three Euclidean basis vectors $\mathbf{e}_i$ all squaring to $+1$, by an additional vector $\mathbf{e}$, also squaring to $+1$, is *identical* to the space being described in the Christian paper in which (eventually), the added vector, here called $\mathbf{e}_\infty$, squares to $+1$. Note this combination of signs means we are dealing with a spherical rather than hyperbolic space.

**Author response**: I reiterate that the geometrical picture constructed in the paper is discussed in considerable detail in Section 2, starting with explaining the necessity of compatifying the physical space $\mathbb{E}^3$ by adding a single point to it at infinity, in order to better understand the origins and strengths of quantum correlations. Compactifying the physical space in this manner transforms it to $S^3$ defined in equation (2.16). While it is well known that the algebraic representation space of $\mathbb{E}^3$ corresponds to the Clifford algebra $Cl_{3,0}$ defined in equation (2.11), the next task in the paper is to find the algebraic representation space of the compactified physical space $S^3$ defined in equation (2.16). After some effort this is then found to be $S^7$ defined in equation (2.60), whose eight-dimensional embedding space happens to be the even subalgebra of $Cl_{4,0}$.

**Reviewer:** So we can understand what Christian is trying to say in the two paragraphs we have picked out so far, as that he is going to use a '1d up' version of CGA, in which the base space points are going to be represented by unit vectors in the higher space, lying on the surface of a sphere, and which can therefore be moved around by the rotors of $Cl_{4,0}$. The extra dimension we need to introduce for this is defined by a basis vector $\mathbf{e}_\infty$ squaring to $+1$, and there is never any question of its squaring to 0.

**Author response**: While I more or less agree with the above summary, it only reflects the mathematical and practical aspects of the constructions presented in the Section 2 of the paper and omits their physical and conceptual aspects, which are essential for understanding the origins and strengths of quantum correlations.

**Reviewer:** The next problem is equation (2.25), which concerns the reversion properties of the pseudoscalar $I_c$, which is introduced in equation (2.24) as

$$I_c = \mathbf{e}_x \mathbf{e}_y \mathbf{e}_z \mathbf{e}_\infty \tag{1.2}$$

The multiplication properties given for the 8 quantities $\{\,1,\,\mathbf{e}_x\mathbf{e}_y,\,\mathbf{e}_z\mathbf{e}_x,\,\mathbf{e}_y\mathbf{e}_z,\,\mathbf{e}_x\mathbf{e}_\infty,\,\mathbf{e}_y\mathbf{e}_\infty,\,\mathbf{e}_z\mathbf{e}_\infty,\,I_c\,\}$ in Table 1, tells us unambiguously that these quantities correspond to the 8 elements of the even subalgebra of $Cl_{4,0}$, with $I_c$ being the pseudoscalar for this space. This is not different from what Christian says, but he says in equation (2.25) that $I_c$ reverses to minus itself, i.e. (to quote)

$$I_c^\dagger \,=\, I_3^\dagger \mathbf{e}_\infty \,=\, -I_3\,\mathbf{e}_\infty \,=\, -I_c\,. \tag{1.3}$$

(Note there appears to be no dispute over the dagger operation being 'reversion', and we will denote it with the usual tilde from now on.)

But this equation is wrong. We have

$$\tilde{I}_c = \mathbf{e}_\infty\mathbf{e}_z\mathbf{e}_y\mathbf{e}_x = -\mathbf{e}_z\mathbf{e}_y\mathbf{e}_x\mathbf{e}_\infty = -\mathbf{e}_y\mathbf{e}_x\mathbf{e}_z\mathbf{e}_\infty = \mathbf{e}_x\mathbf{e}_y\mathbf{e}_z\mathbf{e}_\infty = I_c \tag{1.4}$$

i.e. it reverses to *plus* itself. Thus if Christian's equation (2.25) is used anywhere, it will lead to error.

**Author response**: To begin with, equation (2.25) is not used anywhere in the paper. It is an *intermediate* step, eventually leading to the multiplication table 1 on page 8. As stated by the reviewer, this table does tabulate the multiplication properties given for the 8 quantities $\{\,1,\,\mathbf{e}_x\mathbf{e}_y,\,\mathbf{e}_z\mathbf{e}_x,\,\mathbf{e}_y\mathbf{e}_z,\,\mathbf{e}_x\mathbf{e}_\infty,\,\mathbf{e}_y\mathbf{e}_\infty,\,\mathbf{e}_z\mathbf{e}_\infty,\,I_3\mathbf{e}_\infty\,\}$, and does tell us unambiguously that these quantities correspond to the 8 elements of the even subalgebra of $Cl_{4,0}$. However, the reviewer has overlooked the context within which equation (2.25) appears in the paper. In particular, once again the reviewer seems to have overlooked what has been discussed in Subsection 2.1 prior to the appearance of equation (2.25), and what has been discussed just before equation (2.25). At that stage in the paper we have not yet arrived at the even subalgebra of $Cl_{4,0}$ or Table 1. We are still working within the algebra $Cl_{3,0}$ defined in the equation (2.10). In this algebra the volume form is $I_3 = \mathbf{e}_x\mathbf{e}_y\mathbf{e}_z$, and its reverse is $I_3^\dagger = \mathbf{e}_z\mathbf{e}_y\mathbf{e}_x$. Now, as stated in the paper "this volume form is open and has the topology of $\mathrm{I\!R}^3$. But we can now close it with the null vector $\mathbf{e}_\infty$." In fact, both $I_3 = \mathbf{e}_x\mathbf{e}_y\mathbf{e}_z$ and its reverse $I_3^\dagger = \mathbf{e}_z\mathbf{e}_y\mathbf{e}_x$ are open and have to be closed with the null vector $\mathbf{e}_\infty$ in the light of the discussion in the Subsection 2.1. Now here are two important points: First, both $I_3 = \mathbf{e}_x\mathbf{e}_y\mathbf{e}_z$ and $I_3^\dagger = \mathbf{e}_z\mathbf{e}_y\mathbf{e}_x$ are volume elements within the same space. If one is deemed right-handed, then the other one is left-handed, and if one is deemed outside-in, then the other one is inside-out. But the important point is that both of them are volume elements within the same space. Secondly, both of them must be closed with the same point — *i.e.*, the same null vector $\mathbf{e}_\infty$ — added at infinity, as depicted in figure 2. The resulting closed volume forms are therefore $I_3\mathbf{e}_\infty$ and $I_3^\dagger\mathbf{e}_\infty = -I_3\mathbf{e}_\infty$. This is the origin of the minus sign in the equation (2.25) of the paper mentioned by the reviewer.

But, as noted above, this equation is not used anywhere else in the paper because it is an intermediate step towards arriving at the multiplication table 1 on page 8. The minus sign in equation (2.25) becomes inconsequential as we pass through the discussion in Subsection 2.3 for this goal, since we have left the orientation of the space $\mathcal{K}^\lambda$ unspecified.

**Reviewer:** The next problem is with equations (2.33) and (2.34), which read

$$\begin{aligned}\mathcal{K}^+ &= \mathrm{span}\{\,1,\,\mathbf{e}_x\mathbf{e}_y,\,\mathbf{e}_z\mathbf{e}_x,\,\mathbf{e}_y\mathbf{e}_z,\,\mathbf{e}_x\mathbf{e}_\infty,\,\mathbf{e}_y\mathbf{e}_\infty,\,\mathbf{e}_z\mathbf{e}_\infty,\,I_c\,\}\\ \mathcal{K}^- &= \mathrm{span}\{\,1,\,-\mathbf{e}_x\mathbf{e}_y,\,-\mathbf{e}_z\mathbf{e}_x,\,-\mathbf{e}_y\mathbf{e}_z,\,-\mathbf{e}_x\mathbf{e}_\infty,\,-\mathbf{e}_y\mathbf{e}_\infty,\,-\mathbf{e}_z\mathbf{e}_\infty,\,-I_c\,\}\end{aligned} \tag{1.5}$$

It seems to be important to Christian's later purposes that $\mathcal{K}^+$ and $\mathcal{K}^-$ are different, but as spans of objects which differ just by scalar factors from the same objects in the other set, they are mathematically identical. Presumably Christian means something different from what he has written, but this would have to be explained, using actual mathematics, before one could continue with the paper.

**Author response**: The vector spaces $\mathcal{K}^+$ and $\mathcal{K}^-$ are *not* identical. They differ in their orientations. If one of them (say $\mathcal{K}^+$) is deemed right-handed, then the other one ($\mathcal{K}^-$) is left-handed, and vice versa [4]. I have devoted an entire subsection, Subsection 2.3, to explain and stress the significance of this elementary fact, using mathematical descriptions. See the definition of orientation on page 7 of the paper [4]. On the page 8 of the paper I have written: "It is easy to verify that the bases of $\mathcal{K}^+$ and $\mathcal{K}^-$ are indeed related by an $8 \times 8$ diagonal matrix whose determinant is $(-1)^7 < 0$. Consequently, $\mathcal{K}^+$ and $\mathcal{K}^-$ indeed represent right-oriented and left-oriented vector spaces, respectively, in accordance with our definition of orientation. We can therefore leave the orientation unspecified and write $\mathcal{K}^{\pm}$ as

$$\mathcal{K}^{\lambda} = \text{span}\{\,1,\, \lambda\mathbf{e}_x\mathbf{e}_y,\, \lambda\mathbf{e}_z\mathbf{e}_x,\, \lambda\mathbf{e}_y\mathbf{e}_z,\, \lambda\mathbf{e}_x\mathbf{e}_\infty,\, \lambda\mathbf{e}_y\mathbf{e}_\infty,\, \lambda\mathbf{e}_z\mathbf{e}_\infty,\, \lambda I_3\mathbf{e}_\infty\,\},\;\; \lambda^2 = 1 \iff \lambda = \pm 1." \tag{2.35}$$

Later in the paper $\lambda$ is taken as a hidden variable with real physical consequences for the strong quantum correlations. I do not mean anything different from what I have written in the Subsection 2.3, the title of which is self-explanatory.

**Reviewer:** (Continuing anyway!) the next problem is with the title and initial remarks of Section 2.4 in the Christian paper. The title is 'Representation space $\mathcal{K}^{\lambda}$ remains closed under multiplication' and the initial remarks are 'As an eight-dimensional linear vector space, $\mathcal{K}^{\lambda}$ has some remarkable properties. To begin with, $\mathcal{K}^{\lambda}$ is closed under multiplication.' The title and remarks seem naive — we are dealing with the even subset of the Clifford algebra $Cl_{4,0}$ so what is said here follows immediately from this fact. The properties are hardly remarkable *per se*.

**Author response**: The title of and remarks in the Subsection 2.4 are hardly naïve. The representation space $\mathcal{K}^{\lambda}$ does remain closed under multiplication. And that is indeed a remarkable property for any space, and especially for the space with octonion-like non-trivial multiplication rules. In fact, the simpler quaternionic 3-sphere defined in equation (2.16) also remains closed under multiplication of any number of quaternions despite the non-trivial rules by which they multiply. If we take, say, one million different quaternions from the set (2.16) and multiply them together in any order, then the resulting entity is again a quaternion belonging to the same set. That is quite remarkable. The same property holds also for the elements of the set $\mathcal{K}^{\lambda}$.

More importantly, for a locally causal understanding of quantum correlations (which is of course the central concern of the paper), the above property of the sets (2.16) and $\mathcal{K}^{\lambda}$ embodies the condition of factorizability demanded by Bell, as discussed in the appendices A and B of the paper. This is a vital condition for respecting local causality. Therefore stressing the importance of this property in the title and contents of the Subsection 2.4 is quite appropriate.

**Reviewer:** More serious is what happens next. It is clear from Christian's equation (2.8) that by 'norm' of a general multivector $M$ he means

$$||M|| = \sqrt{\langle M\tilde{M}\rangle} \tag{1.6}$$

**Author response**: The left-hand side of the above equation is — by definition — a scalar number, $||M||$. There are two equivalent ways to work out this scalar number:

(1) $||M||$ = square root of the scalar part $\langle M\tilde{M}\rangle$ of the geometric product $M\tilde{M}$ between $M$ and $\tilde{M}$

or

(2) $||M||$ = square root of the geometric product $M\tilde{M}$ with the non-scalar part of $M\tilde{M}$ set equal to zero.

The above two definitions of the norm $||M||$ are *entirely equivalent*. They give the same scalar value for the norm.

Regardless of GA, $\tilde{M}$ is generally said to be the conjugate of $M$ if $M\tilde{M}$ happens to be equal to unity: $M\tilde{M} = 1$.

It is also important to recall that in Geometric Algebra the fundamental product between any two multivectors $M$ and $N$ is the geometric product, $MN$, *not* the scalar product $\langle MN\rangle$ (or the wedge product $M \wedge N$ for that matter). Therefore, in general, the product that must be used in computing the norm $||M||$ that preserves the Clifford algebraic structure of $\mathcal{K}^\lambda$ is the geometric product $M\tilde{M}$, and not the scalar product $\langle M\tilde{M}\rangle$. To be sure, in practice, if one is interested only in working out the value of the norm $||M||$, then it is often convenient to use the definition (1) above. However, when the primary purpose in working out the norm is to preserve the underlying algebraic structure of the space under consideration in a fundamental relation such as

$$||MN|| = ||M||\,||N||\,,$$

then the definition of the norm that must be used for that purpose is necessarily the second definition stated above.

In the light of these observations it is not difficult to see that the rest of the reviewer comments below are missing a crucial point. For the completeness of my response, however, let me continue to address the comments point-by-point.

**Reviewer:** We can see that this square root is valid, and won't lead to imaginaries, as follows. Let us set up a general $M$ via defining two 2-spinors $\phi$ and $\chi$ as

$$\begin{aligned} \phi &= a_0 + a_1 e_y e_z + a_2 e_z e_x + a_3 e_x e_y \\ \chi &= b_0 + b_1 e_y e_z + b_2 e_z e_x + b_3 e_x e_y \end{aligned} \tag{1.7}$$

and write

$$M = \phi + I\chi \tag{1.8}$$

(Note we are going to write $I_c$ as $I$ from now on). Since $I^2 = 1$ we have

$$M\tilde{M} = \phi\tilde{\phi} + \chi\tilde{\chi} + I\left(\phi\tilde{\chi} + \chi\tilde{\phi}\right) \tag{1.9}$$

Now, let us define two 4-vectors using the components of $\phi$ and $\chi$

$$a = a_0 e_\infty + a_1 e_1 + a_2 e_2 + a_3 e_3$$
$$b = b_0 e_\infty + b_1 e_1 + b_2 e_2 + b_3 e_3 \qquad (1.10)$$

(Note we are not saying that $\phi$ or $\chi$ are 4-vectors. We are just defining objects that make it easy to display the components of $M\tilde{M}$.) Then we find $\phi\tilde{\phi} + \chi\tilde{\chi} = a^2 + b^2$ and $\phi\tilde{\chi} + \chi\tilde{\phi}$ is the scalar $2a \cdot b$, meaning

$$M\tilde{M} = a^2 + b^2 + 2a \cdot bI \qquad (1.11)$$

This shows us that $\langle M\tilde{M}\rangle = a^2 + b^2$ is indeed positive if $M$ is non-zero, hence the norm is well-defined.

**Author response**: Despite the intention, this change in notation introduced by the reviewer does not really add anything enlightening. In fact, it obscures the physical meanings of the relationships between various spaces that are vital in understanding quantum correlations. Moreover, it introduces a subtle form of straw man before knocking it down. The straw man is introduced in equation (1.10) above in the form of the following two arbitrary 4-vectors:

$$a = a_0 e_\infty + a_1 e_1 + a_2 e_2 + a_3 e_3$$
$$b = b_0 e_\infty + b_1 e_1 + b_2 e_2 + b_3 e_3 \,. \qquad (1.10)$$

Since these vectors are introduced in *ad hoc* manner, nothing prevents us from choosing them so that they remain orthogonal to each other: $a \cdot b = 0$. As we shall see, this nullifies some of the claims made by the reviewer below.

**Reviewer:** Given any two general elements $X$ and $Y$, Christian then decides to normalise them, setting

$$||X||^2 = 1, \qquad ||Y||^2 = 1 \qquad (1.12)$$

Christian at this point does not motivate why we would wish to do this, but as just established, it is something we can indeed carry out for any non-zero elements.

**Author response**: The motivation for normalizing the general elements $X$ and $Y$ of $\mathcal{K}^\lambda$ to unity is obvious from the start. It is obvious from the discussions in the abstract, introduction, Section 2, and figure 2, from which it is clear that we are heading towards a compactified physical space $S^3$ and its algebraic representation space $S^7$, which are well known to be two of the only four parallelizable spheres; namely, $S^0$, $S^1$, $S^3$ and $S^7$ (see also Appendix A).

**Reviewer:** So far, so good. However, things go very wrong with equation (2.40). Christian states:

> We shall soon see that for vectors $X$ and $Y$ in $\mathcal{K}^\lambda$ (not necessarily unit) the following relation holds:
>
> $$||XY|| = ||X|| \, ||Y|| \qquad (2.40)$$

(By 'vector' Christian means what we would call 'multivector' here, as is clear from the context.) However, this is false.

**Author response**: The validity of the equation (2.40) of the paper — which is identical to equation (2.59) apart from notation — has been explicitly proved in the paper. The proof uses the second definition of the norm $||M||$:

(2) $||M||$ = square root of the geometric product $M\tilde{M}$ with the non-scalar part of $M\tilde{M}$ set equal to zero.

As I pointed out above, this definition is entirely equivalent to definition (2.8), and it is the correct definition for the norm in the present context, because it preserves the algebraic structure of the space $\mathcal{K}^\lambda$ within the norm relation (2.40). I have also provided further details of the proof in the preprint entitled "Eight-dimensional Octonion-like but Associative Normed Division Algebra": `https://hal.archives-ouvertes.fr/hal-01933757/document`.

**Reviewer:** Consider the quantities

$$I_+ = \frac{1}{2}(1+I), \qquad I_- = \frac{1}{2}(1-I) \tag{1.13}$$

Since $I$ squares to 1 and is its own reverse, then these satisfy the relations

$$I_+^2 = I_+\tilde{I}_+ = I_+\,, \quad I_-^2 = I_-\tilde{I}_- = I_-\,, \quad I_+I_- = I_-I_+ = 0 \tag{1.14}$$

We call such quantities 'idempotents' (since they square to themselves) and this particular pair are 'orthogonal' (since their product is zero). Now let

$$X = \sqrt{2}I_+\,, \quad Y = \sqrt{2}I_-\,, \tag{1.15}$$

These satisfy

$$||X|| = 1, \qquad ||Y|| = 1, \quad \text{but} \quad ||XY|| = 0 \tag{1.16}$$

This disproves the assertion in Christian's (2.40). It also means that the assertion which follows it:

> One of the important observations here is that, without loss of generality, we can restrict our representation space to a set of unit vectors in $\mathcal{K}^\lambda$

is false, since if $||X||$ and $||Y||$ are unit vectors, it does not follow that $Z = XY$ is also a unit vector, despite what Christian says in his equation (2.41).

**Author response**: The reviewer's equation (1.16) is not valid, because the quantities defined in (1.13) contradict the definition (2) of the norm I have discussed above. It is easy to see using the reviewer's own notation that the definition (2) of the norm implies that the quantities defined by the reviewer in (1.16) amounts to assuming $+1 = -1$.

In reviewer's notation the general element of $\mathcal{K}^\lambda$ is written as $M = \phi + I\chi$ [see equation (1.8) above]. Then, given reviewer's equation (1.9) and the definition (2) of the norm I discussed above, the normalization condition is given by

$$\phi\tilde{\chi} + \chi\tilde{\phi} = 0 \iff \phi\tilde{\chi} = -\chi\tilde{\phi}$$

Now consider the quantity $I_+ = \frac{1}{2}(1+I)$ considered by the reviewer. According to the general element $M = \phi + I\chi$ this quantity is obtained by setting setting $\phi = \frac{1}{2}$ and $\chi = \frac{1}{2}$. Substituting these values, together with $\tilde{\phi} = \frac{1}{2}$ and $\tilde{\chi} = \frac{1}{2}$ (because $\phi = \frac{1}{2}$ and $\chi = \frac{1}{2}$ are scalars), into the above normalization condition $\phi\tilde{\chi} = -\chi\tilde{\phi}$ leads to the following contradiction:

$$\frac{1}{2} \times \frac{1}{2} = -\frac{1}{2} \times \frac{1}{2}$$
$$\Longrightarrow +1 = -1.$$

Similarly, consider the quantity $I_- = \frac{1}{2}(1-I)$. According to the reviewer's general element $M = \phi + I\chi$ this quantity is obtained by setting setting $\phi = \frac{1}{2}$ and $\chi = -\frac{1}{2}$. Substituting these values, together with $\tilde{\phi} = \frac{1}{2}$ and $\tilde{\chi} = -\frac{1}{2}$ (because $\phi = \frac{1}{2}$ and $\chi = -\frac{1}{2}$ are scalars), into the above normalization condition $\phi\tilde{\chi} = -\chi\tilde{\phi}$ we again arrive at the following contradiction:

$$-\frac{1}{2} \times \frac{1}{2} = +\frac{1}{2} \times \frac{1}{2}$$
$$\Longrightarrow -1 = +1.$$

This proves, by *reductio ad absurdum*, that the *ad hoc* quantities $I_+ = \frac{1}{2}(1+I)$ and $I_- = \frac{1}{2}(1-I)$ assumed by the reviewer are incompatible with the normalization condition $\phi\tilde{\chi}+\chi\tilde{\phi} = 0$, and therefore the claim made by the reviewer in the equation (1.16) above is not correct. As a result, the rest of the comments by the reviewer are also nullified. In particular, if $X$ and $Y$ are unit vectors, then so is $Z = XY$, as a consequence of the norm relation $||XY|| = ||X||\;||Y||$.

**Reviewer:** In Section 2.5 there is further confusion about a quantity which when first introduced squares to 0, but then later squares to 1. In (2.47) and (2.48) the quantity $\varepsilon$, which satisfies $\varepsilon^2 = 0$ is brought in to allow the definition of *biquaternions*, via

$$\mathbb{Q}_z = \mathbf{q}_r + \mathbf{q}_d\,\varepsilon \tag{1.17}$$

where $\mathbf{q}_r$ and $\mathbf{q}_d$ are quaternions. In equation (2.51), however, $\varepsilon$ is identified with $-I$, and it is stated that $\varepsilon^2 = +1$. Thus the previous reference to biquaternions was a red herring.

**Author response**: As I explained before on pages 4 to 6 of this response, the vector $\mathbf{e}_\infty$ is a null vector in $S^3$ but a unit vector in its embedding space $\mathrm{I\!R}^4$. This is very clearly explained in Subsection 2.1 with figure 2. In equation (2.51) of Subsection 2.5 it is again made clear in parenthesis why $\varepsilon^2 = +1$: "since $\mathbf{e}_\infty$ is a unit vector within $\mathcal{K}^\lambda$." There is no "red herring" regarding the pedagogical reference to biquaternions in the beginning of the Subsection 2.5. Once again, the paper under discussion is a physics paper, not a mathematics paper, requiring some pedagogy.

**Reviewer:** What is actually being introduced is the construction we have used above, where one can write a general element of the even subalgebra of $Cl_{(4,0)}$ as

$$M = \phi + I\chi \tag{1.18}$$

with $\phi$ and $\chi$ as given in equations (1.7). We called these 2-spinors above, but it is fine to identify them as quaternions as well. So this shows that translating the quantities introduced by Christian in this section into our notation, we

have

$$\mathbf{q}_r = \phi, \qquad \mathbf{q}_d = \chi, \qquad \mathbb{Q}_z = \mathbf{q}_r + \mathbf{q}_d\,\varepsilon = M = \phi + I\chi \tag{1.19}$$

**Author response**: As noted above, the new notation introduced by the reviewer does not add anything to the analysis. Instead, it ends up obscuring the physical meanings of the relationships between various spaces that are crucial for understanding quantum correlations.

**Reviewer:** (A slight problem is that since Christian says that $\varepsilon$ is equal to the *reverse* of $I$ and he believes (wrongly) that this is $-I$, some signs will start to get out of drift as regards components of his $\mathbf{q}_d$ quaternion versus our $\chi$, but I don't think this is crucial.)

**Author response**: As I explained on page 7 of this response, equation (2.25) is an *intermediate step*, leading to the multiplication table 1 on page 8. There are no "beliefs" in my paper. Every mathematical step in it is presented clearly, explained lucidly and demonstrated rigorously. In particular, $\varepsilon$ is clearly defined as $-\lambda\, I_3\mathbf{e}_\infty$ in equation (2.51). Ultimately what is important for the computations is the correctness of the multiplication table 1 on page 8.

**Reviewer:** Now we have so far skipped over one feature of the construction of $\mathbb{Q}_z$, which is that Christian wants each of $\mathbf{q}_r$ and $\mathbf{q}_d$ to be normalised, with

$$||\mathbf{q}_r|| = ||\mathbf{q}_d|| = \varrho \tag{1.20}$$

where $\varrho$ is some fixed scalar.

**Author response**: $\mathbf{q}_r$ and $\mathbf{q}_d$ are normalized to radius $\varrho$ because they are taken to be elements of the set (2.16) of normalized quaternions. It appears that the reviewer does not refer at all to the physical problem being addressed in the paper. But once attention is paid to the physical problem being addressed, many mathematical steps taken in the paper become clear without requiring justification at each step. The summary of these steps is stated clearly in the abstract of the paper: "Remarkably, the resulting algebra remains associative, and allows us to understand the origins and strengths of all quantum correlations locally, in terms of the geometry of the compactified physical space, namely, that of a quaternionic 3-sphere, $S^3$, with $S^7$ being its algebraic representation space." The very mentions of $S^3$ and $S^7$ indicates that the quaternions we will be using will have to be normalized.

**Reviewer:** He then correctly says in equation (2.53) that this means

$$||\mathbb{Q}_z|| = \sqrt{2}\varrho \tag{1.21}$$

However, things go very wrong in the next equation. Christian says

> Now the normalization of $\mathbb{Q}_z$ in fact necessitates that every $\mathbf{q}_r$ be orthogonal to its dual $\mathbf{q}_d$

$$||\mathbf{Q}_z|| = \sqrt{2}\varrho \implies \mathbf{q}_r\tilde{\mathbf{q}}_d + \mathbf{q}_d\tilde{\mathbf{q}}_r = 0 \tag{1.22}$$

This is false. The same result in the above notation (as used in our equation (1.11)) would be that

$$a = \varrho \quad \text{and} \quad b = \varrho \quad \Longrightarrow \quad a \cdot b = 0 \tag{1.23}$$

which is patently wrong.

**Author response**: The quantities $a$ and $b$ introduced in equation (1.10) are *vectors*, whereas the radius $\varrho$ is a *scalar*, therefore the above equation with $a = \varrho$ and $b = \varrho$ does not make sense. But I suppose the reviewer meant to write

$$||a|| = \varrho \quad \text{and} \quad ||b|| = \varrho \quad \Longrightarrow \quad a \cdot b = 0. \tag{1.23-Corrected}$$

Now, far from being "patently wrong", $a \cdot b \equiv \mathbf{q}_r \tilde{\mathbf{q}}_d + \mathbf{q}_d \tilde{\mathbf{q}}_r = 0$ is exactly the correct normalization condition stemming from the second definition of the norm discussed on the page 9 of this response. Unfortunately, as noted before, the change in notation introduced by the reviewer introduces a subtle form of straw man. The straw man is introduced in the form of the following two *ad hoc* 4-vectors:

$$\begin{aligned} a &= a_0 e_\infty + a_1 e_1 + a_2 e_2 + a_3 e_3 \\ b &= b_0 e_\infty + b_1 e_1 + b_2 e_2 + b_3 e_3 \, . \end{aligned} \tag{1.10}$$

Therefore the the condition $a \cdot b = 0$ is a straw man and the claim that it is "patently wrong" is a paper tiger. Since these vectors are chosen arbitrarily, nothing prevents us from choosing them so that they remain orthogonal to each other: $a \cdot b = 0$. That, then, is the condition of normalization equivalent to the condition $\mathbf{q}_r \mathbf{q}_d^\dagger + \mathbf{q}_d \mathbf{q}_r^\dagger = 0$ in the paper. In terms of the components of $\mathbf{q}_r$ and $\mathbf{q}_d$ this condition, in turn, is equivalent to the constraint function

$$f_{\mathcal{K}} = q_0 q_7 + q_1 q_6 + q_2 q_5 + q_3 q_4 = 0,$$

which in reviewer's notation is identical to

$$f_{\mathcal{K}} = a_0 b_0 + a_1 b_1 + a_2 b_2 + a_3 b_3 = 0.$$

This constraint simply reduces the space $\mathcal{K}^\lambda$ to the sphere $S^7$, thereby reducing the 8 dimensions of $\mathcal{K}^\lambda$ to the 7 dimensions of $S^7$, when we use the second definition of the norm discussed on the page 9 of this response. But such a dimensional reduction should not surprise anyone. That is exactly how the normed division algebras work. The sets $\mathbb{R}$, $\mathbb{C}$, $\mathbb{H}$, and $\mathbb{O}$ of dimensions 1, 2, 4, and 8 are reduced to the spheres $S^0$, $S^1$, $S^3$, and $S^7$ of dimensions 0, 1, 3, and 7, respectively, facilitated by the norm relation $||XY|| = ||X||\,||Y||$ and aided by a different constraint function $f = 0$ in each of the four cases. Similarly, in my paper the space $\mathcal{K}^\lambda$ of 8 dimensions is reduced to the sphere $S^7$ of 7 dimensions by the above constraint, derived using the geometric product $XX^\dagger$ and the normalization condition $\mathbf{q}_r \mathbf{q}_d^\dagger + \mathbf{q}_d \mathbf{q}_r^\dagger = 0$, as explained on the page 9 of this response. Thus, nothing unusual is being carried out in my paper in this regard. On the other hand, the 7-sphere thus constructed in the paper has a topology that is different from that of the octonionic $S^7$, and that difference is captured by the difference in the corresponding normalization constraints

$$f(q_0, q_1, q_2, q_3, q_4, q_5, q_6, q_7) = 0.$$

More precisely, the two respective normalization constraints are:

$$f_{\mathbb{O}} = q_0^2 + q_1^2 + q_2^2 + q_3^2 + q_4^2 + q_5^2 + q_6^2 + q_7^2 - \rho^2 = 0,$$

which reduces the set $\mathbb{O}$ to the sphere $S^7$ made up of 8D vectors of fixed length (or radius) $\rho$, and

$$f_{\mathcal{K}} = q_0 q_7 + q_1 q_6 + q_2 q_5 + q_3 q_4 = 0,$$

which reduces the set $\mathcal{K}^\lambda$ to the sphere $S^7$ made up of a different collection of 8D vectors of fixed length (or radius) $\rho$. This difference arises because in my paper the fundamental geometric product $XX^\dagger$ is used, as it must be, rather than the non-fundamental scalar product, to derive the constraint $f_{\mathcal{K}} = 0$. But both constraints give an identical result for the norms $||X||$, as I have explained on the page 9 of this response.

**Reviewer:** So can we understand why Christian believes this? Tracing through what happens in the following two equations, it is clear that the mistake is made at the point where he says that it is needed for $\mathbb{Q}_z\tilde{\mathbb{Q}}_z$ to be a scalar. If it were needed then indeed it follows that $2a \cdot b = \mathbf{q}_r\tilde{\mathbf{q}}_d + \mathbf{q}_d\tilde{\mathbf{q}}_r$ would have to vanish, but from what has been said about $\mathbb{Q}_z$ so far there is no such requirement — it has just been required that the norm (as defined by Christian and which we examined above) has to have value $2\varrho$. It looks as though what's happening is that Christian has temporarily forgotten that the norm is just the scalar part of the product $M\tilde{M}$, not both the scalar and grade-4 parts. This is a very important mistake.

**Author response**: I have not forgotten anything, temporarily or otherwise. The norm is indeed just the scalar part of the geometric product $M\tilde{M}$. Please see my previous response.

**Reviewer:** Of course there had to be a significant mistake, since it is needed to be able to prove the (false) assertion about the product of the norms being the norm of the product. This is repeated in terms of $\mathbb{Q}$s in equation (2.59). The 'proof' of this amounts to the fact that if two $\mathbb{Q}$s each individually have vanishing grade-4 part when forming $\mathbb{Q}_z\tilde{\mathbb{Q}}_z$, then the product of their norms is equal to the norm of their product. This is fine, but only applies to this special class of $\mathbb{Q}$s, not the whole even subalgebra of $Cl_{(4,0)}$, as Christian claims.

There is a lot of discussion around this part of Section 2.5, attempting to say that due to the relations proved for the norms, therefore he has discovered a new *associative* version of the normed division algebra hitherto represented by the octonians, but of course this is absolutely false, as it had to be, since the relations he is talking about only apply to a limited subset of the space, not the whole space.

**Author response**: The assertion that the product of the norms is equal to the norm of the product, namely equations (2.40) and (2.59) in the paper, is correct. It has been proved explicitly in the paper, with further details provided in this preprint: `https://hal.archives-ouvertes.fr/hal-01933757/document`. See also my previous two responses. In particular, the reviewer's claim that "This is fine, but only applies to this special class of $\mathbb{Q}$s, not the whole even subalgebra of $Cl_{(4,0)}$, ..." is incorrect, because, as explained above, the dimensional reduction of the eight-dimensional space $\mathcal{K}^\lambda$ to the 7-dimensional $S^7$, aided by the norm relation $||XY|| = ||X||\,||Y||$, is precisely how the normed division algebras work. No one should be surprised by such a dimensional reduction in this context.

**Reviewer:** 1.2. What has been achieved by the end of Section 2 of the Christian paper

This completes a quick survey of the initial problems in the Christian paper, taking us through to the start of the discussion concerning quantum state. Hence this is a good time to set down what the mathematical apparatus Christian has assembled to this point actually amounts to. This can be summarised as follows.

Let us consider the even subalgebra of $Cl_{(4,0)}$ and pick out the elements $R$ from this which satisfy

$$R\tilde{R} = 1$$

i.e. we pick out the set of what are usually called *rotors* in this space. Then all of Christian's working to this point boils down to the result that if $S$ is another rotor, then the combination $SR$ is a further rotor, since it satisfies

$$SR\widetilde{(SR)} = SR\tilde{R}\tilde{S} = 1 \tag{1.24}$$

This is true but hardly very exciting!

**Author response**: These concluding comments are an extraordinary U-turn by the reviewer. In nearly half of the commentary by the reviewer, in three out of seven pages of the commentary (on the pages 4, 5 and 6), the reviewer has attempted to demonstrate that the equation (2.40) [or equivalently the equation (2.59)] of my paper, namely

$$||XY|| = ||X||\,||Y||\,, \tag{2.40}$$

is wrong. But now in the concluding comments the reviewer claims that the above equation is in fact trivially true!

So which is the case? Is equation (2.40) "patently wrong" or trivially true? In fact, it is neither wrong nor trivially true. It is *non-trivially* true. It is instructive to see this in the reviewer's own notation used in equations (1.8) and (1.9). In the above comments the reviewer considers two *normalized* rotors, $R$ and $S$, which can be set up as follows:

$$||R|| = \sqrt{R\tilde{R}} \;= \varrho_{\scriptscriptstyle R}$$
$$\text{and} \qquad ||S|| = \sqrt{S\tilde{S}} \;= \varrho_{\scriptscriptstyle S}\,,$$

where $\varrho_{\scriptscriptstyle R}$ and $\varrho_{\scriptscriptstyle S}$ are some fixed scalars. Then the product $SR$ of these two rotors is another rotor, giving

$$\begin{aligned} ||SR|| &= \sqrt{(SR)\widetilde{(SR)}} \\ &= \sqrt{SR\tilde{R}\tilde{S}} \\ &= \sqrt{S\,\varrho^2_{\scriptscriptstyle R}\,\tilde{S}} \\ &= \left(\sqrt{S\tilde{S}}\,\right)\varrho_{\scriptscriptstyle R} \\ &= \varrho_{\scriptscriptstyle S}\,\varrho_{\scriptscriptstyle R} \\ &= ||S||\,||R||. \end{aligned}$$

Thus it does appear at first sight that for normalized rotors my equation (2.40) is trivially true. But this impression is wrong. Because, in reviewer's notation, we have

$$R = \phi + I\chi \qquad \text{and} \qquad R\tilde{R} = a^2 + b^2 + 2(a\cdot b)I\,,$$

and analogous relations for $S$. Thus to normalize $R$ we have to set $a \cdot b = 0$. That is easy enough to do for both $R$ and $S$, but what is involved in the above derivation is their product $SR$ and its conjugate $\widetilde{SR}$, and that makes the calculation non-trivial. The non-triviality of the calculation stems from the fact that, unlike the seven imaginaries of octonions, there are only six basis elements of $\mathcal{K}^\lambda$ that are imaginary. The seventh, $\lambda I_3 \mathbf{e}_\infty$, squares to $+1$. This is evident from the multiplication table 1 on page 8 of the paper. Since the full calculation is shown in detail in the preprint cited previously and in Ref. [5], there is no point in my repeating the calculation here in reviewer's notation.

Thus, contrary to the reviewer's opinion, the results obtained in Section 2 of the paper are significant. Are they "very exciting"? Indeed they are, but not for the reason mentioned in the previous paragraph. The main point of Section 2 is physical, as summarized in the abstract: "Remarkably, the resulting algebra remains associative, and allows us to understand the origins and strengths of all quantum correlations locally, in terms of the geometry of the compactified physical space, namely, that of a quaternionic 3-sphere, $S^3$, with $S^7$ being its algebraic representation space." The elaborate apparatus I have introduced in the Section 2 of the paper is not only vital for understanding the physical reasons behind the origins and strength of all quantum correlations by means of the interplay between $S^3$ and $S^7$ as demonstrated in the later sections, but also necessary for proving the norm relation (2.40) as *non-trivially* true.

## Concluding Remarks:

In summary, a number of misunderstandings have arisen. But my responses to them above demonstrate that there are no mathematical errors or misstatements in my paper. Publishing this response letter online together with my previous replies to reviewers (which are available from the Review History of the paper [1]) will assist the readers. Therefore I sincerely thank the reviewer for this opportunity to add further clarity to the ideas presented in my work.


[1] J. Christian, *Quantum Correlations are Weaved by the Spinors of the Euclidean Primitives*, R. Soc. Open Sci. 5, 180526; doi:10.1098/rsos.180526 (2018); See also the Review History of this paper (available online with the paper, 2018).

[2] L. Dorst, D. Fontijne and S. Mann, *Geometric Algebra for Computer Science* (Elsevier, Amsterdam, 2007).

[3] J. Christian, *Refutation of Scott Aaronson's Critique of my Disproof of Bell's Theorem*, `https://www.academia.edu/38423874/Refutation_of_Scott_Aaronsons_Critique_of_my_Disproof_of_Bells_Theorem` (2019).

[4] J. W. Milnor, *Topology from the Differentiable Viewpoint* (Princeton University Press, Princeton, New Jersey, 1997).

[5] J. Christian, *Eight-dimensional Octonion-like but Associative Normed Division Algebra*, `https://hal.archives-ouvertes.fr/hal-01933757/document` (2018).

# Appendix C

## 1. Response to comments by Joy Christian

The section and page numbers below need to refer to the sections and pages in the comments made by myself on the Joy Christian paper (i.e. to the text "Some problems with a recent Joy Christian paper"), also to the actual Royal Society paper by JC, and also to the response received from JC, so the context will be made clear in each case.

Joy Christian makes several comments on my introductory paragraphs (which constitute the first page of my document). In the areas where he disagrees, this is either to say that my comments about mathematical errors will be shown to be wrong in his following text, or that I shouldn't be talking about 'quantum mechanics' when referring to various portions of his work, or indeed to Bell's Theorem itself. I don't understand this latter point, but since this is just nomenclature it doesn't seem necessary to pursue this at this stage. Thus I think in terms of the validity of the RS paper, which is the question at hand, the response from JC through to the top part of (his) page 4 does not need a reply, since nothing is changed by it.

We now get to my comments (page 2 of my text) relating to the vector $e_\infty$, and to the issues about it squaring to either 0 or 1 depending on context. JC says that I have misunderstood what is going on here due to skipping his Section 2.1, and that I should look at that section for the definition of $e_\infty$ and for why we should take it as null. In fact his Section 2.1, as far as I can tell, defines (after the redefinition of origin)

$$e_\infty = (0, 1) = \hat{x}_4$$

where from equations (2.13) and (2.14) $\hat{x}_4$ unambiguously squares to $+1$. Thus from this section we would take $e_\infty$ as being a unit magnitude vector, and in fact, despite what JC says in his reply, nothing is mentioned in this section about it being null. The first mention of it being null in fact occurs at the start of (JC's) Section 2.2 where it is said that we will 'take its algebraic counterpart to be a non-zero vector of zero norm'. Reference is then made to the role of null vectors in the standard Conformal Geometric Algebra. Indeed both finite and infinite points are represented by null vectors there, but JC has not introduced the '2d' up space needed for the full CGA to work, only a '1d up' space, with the extra vector in that space, $e_\infty = \hat{x}_4$, squaring to $+1$, so we cannot appeal to the CGA for the reason as to why $e_\infty$ should be taken as null.

Anyway, discussion on this point is not relevant to what happens later in the paper, as acknowledged by JC in his reply, where he says 'The actual calculations of the strong correlations are carried out entirely using the algebra given in the multiplication table 1 on page 8 of the paper, which involves only $e_\infty = +1$'. I agree with this, and it therefore means that all the discussion in the paper about an $e_\infty$ being introduced which is null, and doesn't change the algebraic structure of $\mathbb{E}^3$, is in any case irrelevant.

In practical terms, this view appears to be shared by JC, who in response to my statement:

*So we can understand what Christian is trying to say in the two paragraphs we have picked out so far, as that he is going to use a '1d up' version of CGA, in which the base space points are going to be represented by unit vectors in the higher space, lying on the surface of a sphere, and which can therefore be moved around by the rotors of* $Cl_{4,0}$*. The extra dimension we need to introduce for this is defined by a basis vector* $e_\infty$ *squaring to* $+1$*, and there is never any question of its squaring to 0.*

says in his reply

*While I more or less agree with the above summary, it only reflects the mathematical and practical aspects of the constructions presented in the paper and omits their physical and conceptual aspects, which are essential for understanding the origins and strengths of quantum correlations.*

I have to say that I don't know what the latter part of this statement means. The good news, however, is that by the bottom of page 6 of JC's reply, we are basically in agreement: to repeat, the work to this point in the paper amounts to setting up a

*'1d up' version of CGA, in which the base space points are going to be represented by unit vectors in the higher space, lying on the surface of a sphere, and which can therefore be moved around by the rotors of* $Cl_{4,0}$*.*

---

We now reach the text starting just before equation (1.2) in my previous comments, and which JC starts discussing about 1/3 of the way down page 7 in his reply. My comment was

*The next problem is equation (2.25), which concerns the reversion properties of the pseudoscalar* $I_c$*, which is introduced in equation (2.24) as*

$$I_c = e_x e_y e_z e_\infty \tag{1.1}$$

*The multiplication properties given for the 8 quantities* $\{1, e_x e_y, e_z e_x, e_y e_z, e_x e_\infty, e_y e_\infty, e_z e_\infty, I_c\}$ *in Table 1, tells us unambiguously that these quantities correspond to the 8 elements of the even subalgebra of* $Cl_{4,0}$*, with* $I_c$ *being the pseudoscalar for this space. This is not different from what Christian says, but he says in equation (2.25) that* $I_c$ *reverses to minus itself, i.e. (to quote)*

$$I_c^\dagger = I_3^\dagger e_\infty = -I_3 e_\infty = -I_c \tag{1.2}$$

*(Note there appears to be no dispute over the dagger operation being 'reversion', and we will denote it with the usual tilde from now on.)*

*But this equation is* wrong. *We have*

$$\tilde{I}_c = e_\infty e_z e_y e_x = -e_z e_y e_x e_\infty = -e_y e_x e_z e_\infty = e_x e_y e_z e_\infty = I_c \tag{1.3}$$

*i.e. it reverses to* plus *itself. Thus if Christian's equation (2.25) is used anywhere, it will lead to error.*

JC's reply on his page 7 is too long for me to typeset here, but essentially amounts to saying (a) that there *is* a justification available for saying that

$$\tilde{I}_c = -I_c$$

and that this comes from the null vector $e_\infty$ construction we have just discussed above, and (b) that the equation stating $\tilde{I}_c = -I_c$ (equation (2.25) in the RS paper) is not in fact used anywhere in that paper, so whether it is wrong or right doesn't matter.

Answer (b) is incorrect, since the availability of counterexamples to the central claim of the first sections of the paper — namely to have found a new associative version of the normed division algebra hitherto represented by the octonians — is affected by the reversion properties of $I_c$, which is clear from the examples constructed previously in my first set of comments (page 5). For answer (a), the specific statements on how the null vector $e_\infty$ construction leads to $\tilde{I}_c = -I_c$ are not understandable by me, and of course we have already criticised the null $e_\infty$ construction above. More directly one can point out that the multiplication table given in Table 1 in the JC paper, which we are both agreed upon, unambiguously shows that $\tilde{I}_c = I_c$. That's really the end of the story, and it's no use saying that equation (25) in the RS paper is only used as an 'intermediate step' in achieving this table (as stated in the JC reply), since the table itself proves the opposite.

---

We next come to my comment:

*The next problem is with equations (2.33) and (2.34), which read*

$$\begin{aligned} \mathcal{K}^+ &= \mathrm{span}\{1, e_x e_y, e_z e_x, e_y e_z, e_x e_\infty, e_y e_\infty, e_z e_\infty, I_c\} \\ \mathcal{K}^- &= \mathrm{span}\{1, -e_x e_y, -e_z e_x, -e_y e_z, -e_x e_\infty, -e_y e_\infty, -e_z e_\infty, -I_c\} \end{aligned} \tag{1.4}$$

*It seems to be important to Christian's later purposes that $\mathcal{K}^+$ and $\mathcal{K}^-$ are different, but as spans of objects which differ just by scalar factors from the same objects in the other set, they are mathematically identical. Presumably Christian means something different from what he has written, but this would have to be explained, using actual mathematics, before one could continue with the paper.*

JC's response to this is to say that I'm wrong and that he means exactly what he says. Of course I accept that bases can have an orientation. What I'm saying, and which JC does not address, is that $\mathcal{K}^+$ and $\mathcal{K}^-$ are defined via the 'span' operation, and mathematically this is defined as the set of quantities arrived at by taking linear combinations (with scalar coefficients) of the objects in the list that the span operates on. So, as spans of objects which differ just by scalar factors from the same objects in the other set, $\mathcal{K}^+$ and $\mathcal{K}^-$ are mathematically identical. This is not addressed, and my comment still stands.

---

My next comment is:

> *the next problem is with the title and initial remarks of Section 2.4 in the Christian paper. The title is 'Representation space $\mathcal{K}^\lambda$ remains closed under multiplication' and the initial remarks are 'As an eight-dimensional linear vector space, $\mathcal{K}^\lambda$ has some remarkable properties. To begin with, $\mathcal{K}^\lambda$ is closed under multiplication.' The title and remarks seem naive — we are dealing with the even subset of the Clifford algebra $Cl_{4,0}$ so what is said here follows immediately from this fact. The properties are hardly remarkable per se.*

JC's response is:

> *The title of and remarks in the Subsection 2.4 are hardly naive. The representation space $\mathcal{K}^\lambda$ does remain closed under multiplication. And that is indeed a remarkable property for any space, and especially for the space with octonion-like non-trivial multiplication rules. In fact, the simpler quaternionic 3 -sphere defined in equation (2.16) also remains closed under multiplication of any number of quaternions despite the non-trivial rules by which they multiply. If we take, say, one million different quaternions from the set* (2.16) *and multiply them together in any order, then the resulting entity is again a quaternion belonging to the same set. That is quite remarkable. The same property holds also for the elements of the set $\mathcal{K}^\lambda$.*
>
> *More importantly, for a locally causal understanding of quantum correlations (which is of course the central concern of the paper), the above property of the sets* (2.16) *and $\mathcal{K}^\lambda$ embodies the condition of factorizability demanded by Bell, as discussed in the appendices A and B of the paper. This a vital condition for respecting local causality. Therefore stressing the importance of this property in the title and contents of the Subsection 2.4 is quite appropriate.*

I'm afraid the first paragraph of this response displays exactly the same problem as I was alluding to. We are dealing with the even subalgebra of a Clifford algebra. This is closed automatically, and there really isn't anything else to say.

As regards the second paragraph, the link with Bell's theorem and possible importance of the closure in relation to this has not been made yet, so the actual text in the paper still looks naive and odd, despite what the author says.

---

The above have all been preliminaries, but now we get to the most important aspect of where JC goes wrong. This is as concerns the norm of quantities within the even subalgebra, and the question of whether his relation (2.40):

$$||XY|| = ||X||\,||Y|| \qquad \textit{(2.40)}$$

holds for all elements $X$ and $Y$ or not. The parts of JC's response which are relevant to this begin at the top of his page 9, and in various forms go through to page 17 (the end of his document). Thus we will not attempt to set out here a point by point rebuttal, instead, I will describe the situation as I see it, which I think is fairly simple, and which a careful reading of JC's comments leads me to.

(As an aside, there is a misprint in equation (1.23) on page 6 of my first set of comments. There should be some norm signs around the $a$ and $b$ vectors which appear in the first part of this equation, so that it should read

$$||a|| = \varrho \quad \text{and} \quad ||b|| = \varrho \implies a{\cdot}b = 0$$

instead of

$$a = \varrho \quad \text{and} \quad b = \varrho \implies a{\cdot}b = 0$$

I thank JC for pointing this out, and am glad that he was nevertheless able to interpret what it should have said.)

An important aspect of what seems to be going on occurs at the top of JC's comments page 9, where in response to my statement that 'it is clear from Christian's equation (2.8) that by 'norm' of a general multivector $M$ he means'

$$||M|| = \sqrt{\langle M\tilde{M}\rangle}$$

JC says

*The left-hand side of the above equation is — by definition — a scalar number, $||M||$. There are two equivalent ways to work out this scalar number:*

*(1) $||M||$= square root of the scalar part $\langle M\tilde{M}\rangle$ of the geometric product $M\tilde{M}$ between $M$ and $\tilde{M}$*

*(2)* $\|M\|$= *square root of the geometric product* $M\tilde{M}$ *with the non-scalar part of* $M\tilde{M}$ *set equal to zero.*

*The above two definitions of the norm* $\|M\|$ *are entirely equivalent. They give the same scalar value for the norm.*

One has to agree that both of these are identical procedures, as stated by JC. (Basically $M\tilde{M}$ has grade 0 and grade 4 parts in general, so to say we can either take the scalar part, or the part that is left when the grade 4 part is discarded, is to say the same thing in each case.) However, shortly afterwards, he says:

*However, when the primary purpose in working out the norm is to preserve the underlying algebraic structure of the space under consideration in a fundamental relation such as*

$$\|MN\| = \|M\|\|N\|$$

*then the definition of the norm that must be used for that purpose is necessarily the second definition stated above.*

Thus having earlier said that the two definitions are entirely equivalent, he now says that they are not, and that for his purposes we have to take definition (2). Having puzzled over this for some while, I think I now understand where the confusion comes from. JC believes that under definition (2) we should not just *discard* the grade 4 part of $M\tilde{M}$, but actively set it to 0. This introduces a constraint on the type of $M$ which we can take the norm of, and constrains $M$ to in fact be a 'scaled rotor', i.e. something which satisfies

$$M\tilde{M} = \rho$$

where $\rho$ is a positive scalar. With this restriction, i.e. that all the quantities $X$, $Y$, $Z$, $\mathbb{Q}_z$ etc. in JC's sections (2.4) and (2.5) (original RS paper), are scaled rotors, then what he has written becomes in the main correct mathematically. In this case, the whole content of the first part of his paper, through to the beginning of Section 3, reduces to exactly to what I said in my first set of comments, i.e.

*Let us consider the even subalgebra of* $Cl_{(4,0)}$ *and pick out the elements* $R$ *from this which satisfy*

$$R\tilde{R} = 1$$

*i.e. we pick out the set of what are usually called* rotors *in this space. Then all of Christian's working to this point boils down to the result that if* $S$ *is another rotor, then the combination* $SR$ *is a further rotor, since it satisfies*

$$SR\widetilde{(SR)} = SR\tilde{R}\tilde{S} = 1$$

Christian himself seems to agree with this assessment towards the end of his responses, since he says I have carried out a U-turn in making this statement, and that I now clearly now support what he is saying.

*However,* the price for this restriction in terms of the aims of JC is heavy. In particular, despite what it explicitly says in Section 2.4 of his paper, the relation

$$||XY|| = ||X||\,||Y||$$

does not apply to all $X$ and $Y$ in $\mathcal{K}^\lambda$, but only to an $X$ and $Y$ which are scaled rotors. Of course we knew it could not apply to all of $\mathcal{K}^\lambda$ since in my previous comments I provided an explicit counterexample. JC says my counterexample is not valid because it ignores the form of norm he is using, which cannot be used with the elements in my counterexample. But as we have seen, this is equivalent to saying that $X\tilde{X}$ and $Y\tilde{Y}$ both have zero grade 4 parts, and so $X$ and $Y$ are indeed scaled rotors.

Now it is a fact that the scaled rotors, while they form a group under composition (basically multiplication from the left), they do not form a group under addition. If we add two of them with some scalar coefficients, then the resulting object when multiplied with its reverse will in general have a grade-4 part, meaning it is no longer a scaled rotor. This kills off any hope that the set of such objects can form a normed division algebra, as claimed.

The 'scaled' bit of the scaled rotors is removed by JC by making everything have a norm of 1. This means that all his quantities, are in fact rotors, which belong to a 6-dimensional subspace. (That this is 6d can be seen independently from the fact that all rotors are exponentials of bivectors in $Cl_{4,0}$, and the bivector space is 6-dimensional.) This means that his claims that he is working with a 7-dimensional subspace of the 8-dimensional even subalgebra of $Cl_{4,0}$ is not correct, and the link with $S^7$, and everything attached to this (Hopf fibration, parallelizability, and the group $E_8$) all vanish.

JC seems partially aware of some of this in his comments made near the bottom of page 14 and top of page 15 of his responses. But the counting doesn't seem correct. Because we are imposing the additional constraint that the grade 4 part of $X\tilde{X}$ vanishes, then the relation deduced from the scalar part equalling one, which would on its own correspond to $S^7$, now drops down the dimension of the space to 6, the dimension of the rotor space, and we now no longer have an $S^7$. I am not an expert on differential topology, or on $S^7$ itself, and JC seems content in his comments (bottom of page 14 and top of page 15) that indeed an extra constraint is introduced relative to what it would take for a pure $S^7$, so maybe I am missing something. However, in an unambigous fashion we can contrast the situation here with that which operates for $S^3$, and its relation to the quaternions. There, if $Q$ is a quaternion (the set of which are just the even subalgebra of $Cl_{3,0}$), then $Q\tilde{Q}$ automatically has only a scalar part, we don't need to artificially set any other part to 0, and so there is an automatic match to $S^3$. As we have seen, this same type of match does not occur for the even subalgebra of $Cl_{4,0}$, since while the scalar part of $X\tilde{X}$ sets up a nice match with $S^7$, the extra

constraint from $\langle X\tilde{X}\rangle_4 = 0$ reduces the overall dimension down to 6, and we are working just with the rotor group.

It is necessary to state that none of this is brought out or stated in the Royal Society paper itself. There it categorically states that

$$||XY|| = ||X||\,||Y||$$

applies to *all* of the even subalgebra of $Cl_{4,0}$, which if true would make it a genuine normed division algebra, but of course we have seen that this is false.

**Conclusion:**

The responses from JC have made it clearer what some of the motivation is, and explained some aspects in a clearer way. However, unfortunately all my previous objections remain, and the end result is that Sections 1 and 2 of the paper succeed only in showing that the set of rotors of $Cl_{4,0}$ (i.e. even elements of $Cl_{4,0}$ which satisfy $M\tilde{M} = 1$) are closed under multiplication. This is a trivial result which can be established in only a few lines. All the associated statements about normed division algebras, oriented bases, Hopf fibrations, $S^7$, $E_8$ etc., are irrelevant and unsubstantiated. Moreover, the paper itself gives no inkling that the restriction to rotors is being applied and indeed stresses the applicability of crucial formulae, such as (2.40), to *all* members of $Cl_{4,0}$, which is simply false. In the responses from JC, this restriction to (scaled) rotors is acknowledged, but specious arguments are given that somehow the 'norm' itself makes this restriction. This makes no sense at all, and it is hard to believe that the distinction between, on the one hand, *discarding* a part of a product, and on the other *actively setting it to zero*, thereby setting an extra constraint, is not understood.

Note it is possible that all JC needs in the second part of the paper, beginning Section 3, is the reduced result concerning scaled rotors I have just described. I haven't been through this part as yet and can't comment on this aspect. However, if so, this part would need to be preceded by a first part of the paper which is completely different from the first part of the current one, to be capable of publication.

# Appendix D

As far as I can judge in the use of geometric algebra by C. Joy, there seem no mathematical algebraical mistakes in using it for modeling. Even though certainly I have not reproduced all computations in full detail. As for the side of physics regarding the Bell inequalities, Joy proposes a different approach from the current main stream of quantum physics interpretation. This is certainly interesting for stimulating fresh discussions, and rethinking the fundamental assumptions involved. He suggests an experiment (reference [17]), that could be a test for his theory. I think this experiment should be done, that would clarify if Joy's new approach is right, or if his critics are right. I think the editor in charge, who admitted Joy's paper for publication must have had to make a difficult decision. But I think it is right to include non-mainstream approaches in Royal Society Open Science, because it can bring fresh life to stale situations in science. And only because something has been published in RSOS, it does not necessarily have to be confirmed by experiment. On the other hand, not publishing a paper like Joy's, I would see as a loss to freedom of opinion in science.

# Appendix E

Author response Rev 2

I thank the reviewer for this positive review
